# The Threshold of Protection from Liver-Stage Malaria Relies on a Fine Balance between the Number of Infected Hepatocytes and Effector CD8^+^ T Cells Present in the Liver

**DOI:** 10.4049/jimmunol.1601209

**Published:** 2017-01-13

**Authors:** Alexandra J. Spencer, Rhea J. Longley, Anita Gola, Marta Ulaszewska, Teresa Lambe, Adrian V. S. Hill

**Affiliations:** The Jenner Institute, University of Oxford, Oxford OX3 7DQ, United Kingdom

## Abstract

Since the demonstration of sterile protection afforded by injection of irradiated sporozoites, CD8^+^ T cells have been shown to play a significant role in protection from liver-stage malaria. This is, however, dependent on the presence of an extremely high number of circulating effector cells, thought to be necessary to scan, locate, and kill infected hepatocytes in the short time that parasites are present in the liver. We used an adoptive transfer model to elucidate the kinetics of the effector CD8^+^ T cell response in the liver following *Plasmodium berghei* sporozoite challenge. Although effector CD8^+^ T cells require <24 h to find, locate, and kill infected hepatocytes, active migration of Ag-specific CD8^+^ T cells into the liver was not observed during the 2-d liver stage of infection, as divided cells were only detected from day 3 postchallenge. However, the percentage of donor cells recruited into division was shown to indicate the level of Ag presentation from infected hepatocytes. By titrating the number of transferred Ag-specific effector CD8^+^ T cells and sporozoites, we demonstrate that achieving protection toward liver-stage malaria is reliant on CD8^+^ T cells being able to locate infected hepatocytes, resulting in a protection threshold dependent on a fine balance between the number of infected hepatocytes and CD8^+^ T cells present in the liver. With such a fine balance determining protection, achieving a high number of CD8^+^ T cells will be critical to the success of a cell-mediated vaccine against liver-stage malaria.

## Introduction

Since the year 2000, the substantial increases in funding and global effects in prevention and treatment of malaria have led to a 40% reduction in clinical disease (1). Despite these efforts, malaria continues to cause significant mortality and morbidity worldwide, with around half a million deaths in 2015 attributed to malaria, with 70% of these occurring in children under the age of 5 y (2). Malaria infection of a mammalian host begins with the release of sporozoites into the skin from the bite of an infected mosquito (3). Within minutes, sporozoites are able to migrate from the dermis to the liver where they infect hepatocytes (4) and undergo asexual replication, leading to release of many thousands of merozoites directly into the bloodstream and infection of RBCs (5). The pre-erythrocytic stage of malaria is nonpathogenic and clinically silent, lasting 6 d in humans (6) but only 2 d in rodents (7). Our knowledge of the adaptive immune response to this stage of infection in humans is limited, as there are no systemic signs of immune reactivity (8) and only low-level immune responses to pre-erythrocytic Ags have been observed in malaria-exposed individuals (9–12).

In the 1970s complete protection from malaria sporozoite challenge was demonstrated in humans (13), similar to rodents (14), by inoculation with irradiated sporozoites. During the following years a number of pivotal studies demonstrated the importance of CD8^+^ T cells in mediating protection (15, 16). This opened the door to vaccination strategies aimed at inducing liver-stage specific CD8^+^ T cells, such as vectored vaccines, irradiated sporozoites, or genetically attenuated parasites.

CD8^+^ T cell–mediated protection of BALB/c mice against *Plasmodium berghei* has been mapped down to a single epitope, Pb9, from the immunodominant Ag, the circumsporozoite protein (17). After initial demonstration that adoptive transfer of Pb9-specific cells was sufficient to achieve protection (17), increasing efficacy of subunit vaccines has been demonstrated in mice with vaccination regimens that induce higher numbers of Pb9-specific cells, whether from the native protein (18–20) or expressed in an epitope string (21, 22). More recently, protection from *Plasmodium falciparum* in humans vaccinated with viral vectors has been shown to correlate with the frequency of circulating Ag-specific CD8^+^ T cells (23). However, to achieve efficacy in both rodents and humans, high number of circulating cells are required (24), with even higher numbers required in rodents than in humans (23, 24).

Despite years of research, very little is still known about how CD8^+^ T cells are reactivated and mediate protection in the liver. Although a number of elegant studies have investigated factors that influence the priming of protective CD8^+^ T cell responses (25–30), it is still not clear why such high numbers of T cells are required for protection. Because only a small fraction of injected sporozoites successfully locate blood vessels and migrate to the liver (31, 32), where parasites are only present for a short period of time (7), one could hypothesize that extremely high numbers of CD8^+^ T cells are required to enable efficient scanning of the small number of infected hepatocytes. Although Kupffer cells and hepatocytes both have the capacity to activate CD8^+^ T cells (33), which cells presents Ag to reactivate CD8^+^ T cells in the context of a sporozoite challenge and how this impacts on protection remain unclear.

In this study we developed an adoptive transfer model to track Ag-specific effector cells in the liver of mice in response to sporozoite challenge. Using viral vectors expressing Pb9, we were able to CFSE label Pb9 effector CD8^+^ T cells and track cell movement and division in recipient mice after sporozoite challenge. By using a vaccination strategy known to induce a protective CD8^+^ T cell phenotype (34) and a natural *P. berghei* Ag, we were able to track native sporozoite Ag presentation in the context of a liver-stage infection. In this study, we show that recruitment of CD8^+^ T cells into division and the presence of dividing CD8^+^ T cells can be used as a marker of Ag presentation in the liver. By altering the number of transferred cells and sporozoites, we identify a threshold in reactivation of CD8^+^ T cells, which significantly impacts protection from liver-stage malaria.

## Materials and Methods

### Ethics statement

Mice were used in accordance with the UK Animals (Scientific Procedures) Act under project license number 30/2414 or 30/2889 granted by the UK Home Office. Animals were group housed in individually vented cages under specific pathogen-free conditions, with constant temperature and humidity and with lighting on a 12:12 light/dark cycle (8 am to 8 pm). For induction of short-term anesthesia, animals were anesthetized using vaporized isoflurane (IsoFlo). All animals were humanely sacrificed at the end of each experiment by an approved schedule 1 method.

### Animals and immunizations

Female BALB/cOlaHsd (BALB/c) mice 6 wk of age or older (Envigo, formally Harlan Laboratories, Bicester, U.K.) were immunized i.m. in the musculus tibialis with 10^8^ infectious units of human adenovirus serotype 5 (HAd5) expressing model Ag TIP–enhanced GFP (eGFP) and at least 6 wk later boosted intradermally (i.d.) in the ear with 10^6^ PFU of modified vaccinia Ankara (MVA) expressing TIPeGFP. In instances of failed sporozoite batches, the interval between HAd5 and MVA immunization was increased or mice were boosted a second time with 10^6^ PFU of MVA. Cell transfers were always performed 10 d after MVA boost.

To disrupt MHC class I presentation (35) and block T cell function, mice were injected i.p. with 100 μl of 0.03 mg/ml FK-506 monohydrate (tacrolimus) (Sigma-Aldrich) to achieve an in vivo dose of 0.15 mg/kg. Due to time-restricted access at the animal facility, FK-506 was administered every morning and evening for the first 2 d at 0, 9, 24, and 32 h after sporozoite injection. For depletion of Kupffer cells, mice received 200 μl of clodronate or PBS liposomes (Clodronate.Liposomes.org) via the i.v. route for 2 d prior to sporozoite administration. Alternatively, phagocytic activity of Kupffer cells was inhibited by injection 10 mg/kg (100 μl of 2 mg/ml) gadolinium chloride (Sigma-Aldrich) the day prior to sporozoite injection (36).

### Vaccines and Ags

The synthetic epitope string TIPe-GFP (37) contains the H-2K^d^–restricted *P. berghei* circumsporozoite protein CD8^+^ T cell epitope Pb9 SYIPSAEKI (aa 252–260) (17) and H-2K^d^–restricted CD8^+^ T cell epitope GFP_200–214_ HYLSTQSAL (38) (peptides purchased from ProImmune). HAd5 and MVA viral vectors containing the TIPeGFP or GFP insert were generated, produced, and titered as previously described (22).

### Cell transfer

Splenocytes from HAd5-MVA–vaccinated mice were treated with ammonium chloride (ACK buffer) to lyse RBCs prior to labeling with 5 μM CFSE for 10 min at 37°C. A small sample of cells was stained with anti–Pb9-allophycocyanin tetramer (obtained through the National Institutes of Health Tetramer Facility) (Supplemental Fig. 1A), CD8-PerCP-Cy5.5 (clone 53-6.7; eBioscience), and CD4–eFluor 450 (clone GK1.5; eBioscience) to determine the frequency of Pb9^+^CD8^+^ T cells prior to cell transfer (Supplemental Fig. 1B). Typically, recipient mice received 3 × 10^6^ CFSE^+^Pb9^+^ splenocytes (∼3–5 × 10^7^ total splenocytes) injected i.v. the day before sporozoite injection. Because effector Pb9 T cells could not successfully be enriched by either negative or positive bead selection (Supplemental Fig. 1C), transferring unfractionated splenocytes reduced the number of vaccinated donor mice required to achieve sufficient numbers of Pb9 cells in these experiments, while also enabling the inclusion of GFP-specific CD8^+^ effector cells as an internal control.

### Malaria sporozoite challenge

*P. berghei* (ANKA strain, clone 234) sporozoites expressing mCherry (provided by Volker Huessler) (39) were isolated from salivary glands of female *Anopheles stephensi* mosquitoes at ∼21 d after feeding on *P. berghei* blood stage–infected donor mice. Salivary glands were homogenized, sporozoites counted under phase-contrast microscopy, and 1000 *P. berghei* sporozoites were injected via the i.v. route into recipient mice. Mice were monitored daily from day 5 onward by taking a thin blood film and staining with 5% Giemsa (Sigma-Aldrich) to screen for the presence of schizonts within the RBCs. Parasitemia was calculated as the percentage of infected RBCs per microscope field (×100 objective), with at least five fields counted per mouse per day. Using linear regression, the time to 0.5% parasitemia was calculated based on the *y*-intercept and slope of the line. Mice were humanely sacrificed by an approved schedule 1 method after three consecutive positive blood films or parasitemia >1%. Sterile protection was defined as the complete absence of parasite in the blood until at least day 8.

### Liver lymphocyte isolation

Mice were sacrificed and livers perfused in situ by injecting a 29-gauge butterfly syringe into the portal vein and flushing with at least 10 ml of Dulbecco’s PBS (Sigma-Aldrich) prior to dissecting out livers and removing the gall bladder. Single-cell suspensions were prepared by passaging livers through 70-μm cell strainers prior to enrichment for lymphocytes by resuspension in a 33% isotonic Percoll solution (Sigma-Aldrich) and centrifugation at 693 × *g* for 12 min (40). Any remaining RBCs were removed by treatment for 5 min with ACK before final resuspension in complete media and stimulating cells for intracellular cytokine staining.

### Intracellular cytokine staining

Single-cell suspensions from spleens and lymph nodes were prepared by passing cells through 70-μm cell strainers and ACK lysis (spleens only). Cells were stimulated at 37°C for 6 h with 1 μg/ml Pb9 peptide or 2 μg/ml GFP_200–208_ peptide or media together with 1 μg/ml GolgiPlug (BD Biosciences) with the addition of 2 μl/ml CD107a-PE (clone eBio1D4B; eBioscience) for the culture period when investigated. Following surface staining for CD4–eFluor 450 and CD8-PerCP-Cy5.5, other surface markers (eBioscience or BioLegend), and Live/Dead Fixable Aqua Dead cell stain (Invitrogen), cells were fixed with 10% neutral buffered formalin solution (containing 4% formaldehyde) (Sigma-Aldrich) for 5 min on ice prior to intracellular staining for cytokines with Abs diluted in Perm/Wash buffer (BD Biosciences). Sample acquisition was performed on an LSR II (BD Biosciences) and data were analyzed with FlowJo v9 (Tree Star). An acquisition threshold was set at a minimum of 5000 events in the live CD8^+^ gate. Pb9-specific cells were identified by gating on Live/Dead negative, doublet negative (forward scatter–height versus forward scatter–area), size (forward scatter–height versus side scatter), CD8^+^CD4^−^ cells, and IFN-γ^+^ (Supplemental Fig. 1D). Cytokine-positive responses are presented after subtraction of the background response detected in the corresponding unstimulated control sample for each mouse. All graphs and statistical analyses were performed using Prism v6 (GraphPad Software) with final figures created in Adobe Illustrator CS5 v15. Recruitment into division was calculated by dividing the number of cells in each division by 2*^n^*, where *n* is equal to the division number. This value indicates the number of original cells that went through at least one cycle of cell division, and is expressed as a percentage of the calculated number of original cells (Supplemental Fig. 1C) (41).

## Results

### Kinetics of the CD8^+^ T cell recall response to liver-stage malaria

**FIGURE 1.**
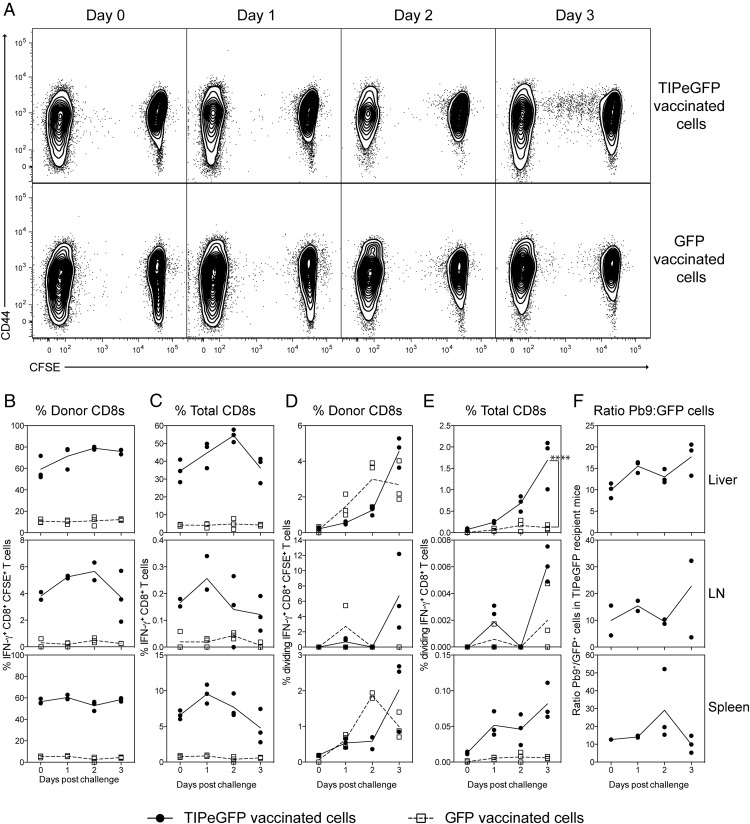
Kinetics of the liver-stage response to malaria. CFSE-labeled splenocytes (1 × 10^7^) from HAd5-MVA TIPeGFP or GFP-vaccinated mice were transferred into BALB/c mice 1 d prior to i.v. challenge with 1000 *P. berghei* sporozoites. Mice were sacrificed before challenge (day 0) and 1, 2, and 3 d postchallenge to analyze the response in the livers and liver-draining lymph nodes (celiac and portal) by flow cytometry. Cells were stimulated with Pb9 or GFP peptide for 6 h prior to surface staining with Live/Dead Aqua, CD8–Pacific Blue, CD62L-PerCP-Cy5.5, CD127–bi av-qDot 565, CD4–eFluor 450, CD25–Alexa Fluor 700, CD44-allophycocyanin-Cy7, and intracellular staining for IL-2–PE, TNF-α–PE-Cy7, and IFN-γ–allophycocyanin. Representative dot plots (**A**) show CD44 versus CFSE expression of all CD8^+^ T cells isolated from the liver after sporozoite challenge. Owing to the low frequency of dividing cells observed, the Pb9, GFP, and media fcs file from each mouse were pooled together (concatenated) for presentation. Graphs represent the frequency of Pb9 IFN-γ–producing CD8^+^ T cells from TIPeGFP-vaccinated mice (●) or GFP IFN-γ–producing CD8^+^ T cells (□) from GFP-vaccinated mice as a percentage of donor CFSE^+^CD8^+^ T cells (**B**) or total CD8^+^ T cells (**C**), the frequency of dividing cells as a percentage of donor CFSE^+^CD8^+^ T cells (**D**), or total CD8^+^ T cells (**E**) isolated from the liver (top panel), liver-draining lymph node (middle panel), or spleen (bottom panel). (**F**) The graph represents the ratio of IFN-γ^+^ Pb9 to IFN-γ^+^ GFP cells in each organ only in the TIPeGFP recipient mice. Each animal is represented by a single point and the mean per group is represented by the line; data are representative of two similar experiments.

To determine the kinetics of the Ag-specific CD8^+^ T cell response to liver-stage malaria infection, splenocytes from viral vector vaccinated mice were CFSE labeled and transferred into recipient mice prior to i.v. challenge with 1000 sporozoites. To induce a high number of Ag-specific (Pb9 or eGFP) CD8^+^ T cells with a protective phenotype (22, 42), donor mice were vaccinated with a heterologous prime boost vaccination regimen comprising HAd5 followed at least 6 wk later by a MVA vaccination with vectors expressing either TIPeGFP (an epitope string containing Pb9 fused to eGFP) or GFP, and cells were transferred 10 d after the MVA boost. Transferring unfractionated splenocytes enabled the transfer of malaria-specific CD8^+^ T cells (Pb9^+^) relative to a nonmalaria CD8^+^ T cell population (eGFP^+^). Additionally, effector cells are not enriched through the standard CD8 negative or positive bead selection process (Supplemental Fig. 1), and therefore by transferring whole splenocytes we were able to transfer large numbers of unmanipulated effector cells. Recipient mice were harvested on the day of challenge and days 1, 2 and 3 after challenge, and the responses in the liver and liver-draining lymph nodes (celiac and portal) (43, 44) and spleen were measured by intracellular cytokine staining (ICS). On the days following sporozoite challenge, marginal (not significant) changes in the frequency of donor IFN-γ^+^ Pb9-specific cells were observed in the liver of mice, whereas very few Pb9-specific cells were observed in the liver-draining lymph nodes or spleen. A small proportion of Pb9-specific dividing cells was observed in the liver at day 2, but a distinct population of dividing cells was not observed in the liver until 3 d postchallenge (Fig. 1A), corresponding to a significant increase in the percentage of dividing IFN-γ^+^ Pb9-specific cells in TIPeGFP recipient mice compared with IFN-γ^+^ GFP-specific cells in GFP recipient controls (Fig. 1) or IFN-γ^+^GFP^+^–specific cells in the same TIPeGFP recipients (data not shown). At this time no dividing cells (<0.01%) were detected in the liver-draining node or spleen (Fig. 1D, 1E), suggesting that the liver is the main site of CD8^+^ T cell reactivation during a liver-stage infection. The small increase in the frequency of Pb9-specific cells was not observed at the total organ level, as there was no significant difference in the total number of Ag-specific Pb9 or GFP cells over time in any organ (data not shown). To normalize for quality of the liver preparations and ICS stimulations and staining performed on separate days, the ratio of Pb9-specific to GFP-specific cells within TIPeGFP recipient mice was used to determine whether there was a relative increase in Pb9-specific cells in the liver over time. Although the ratio of Pb9-specific to GFP-specific cells increased over time, no statistically significant difference between days was observed, suggesting that Pb9 CD8^+^ T cells were not actively migrating into the liver during liver-stage infection. When the same experiment was performed with sporozoites administered via the i.d. route, very few dividing cells were detected in the liver before day 7 (Supplemental Fig. 2). As <1% of sporozoites have been predicted to reach the liver after i.d. injection, the absence of dividing cells in the liver before day 7 could be an indication of the overall low Ag (malaria) liver load.

### Ag-specific CD8^+^ T cells do not actively migrate into the liver during a malaria infection

**FIGURE 2.**
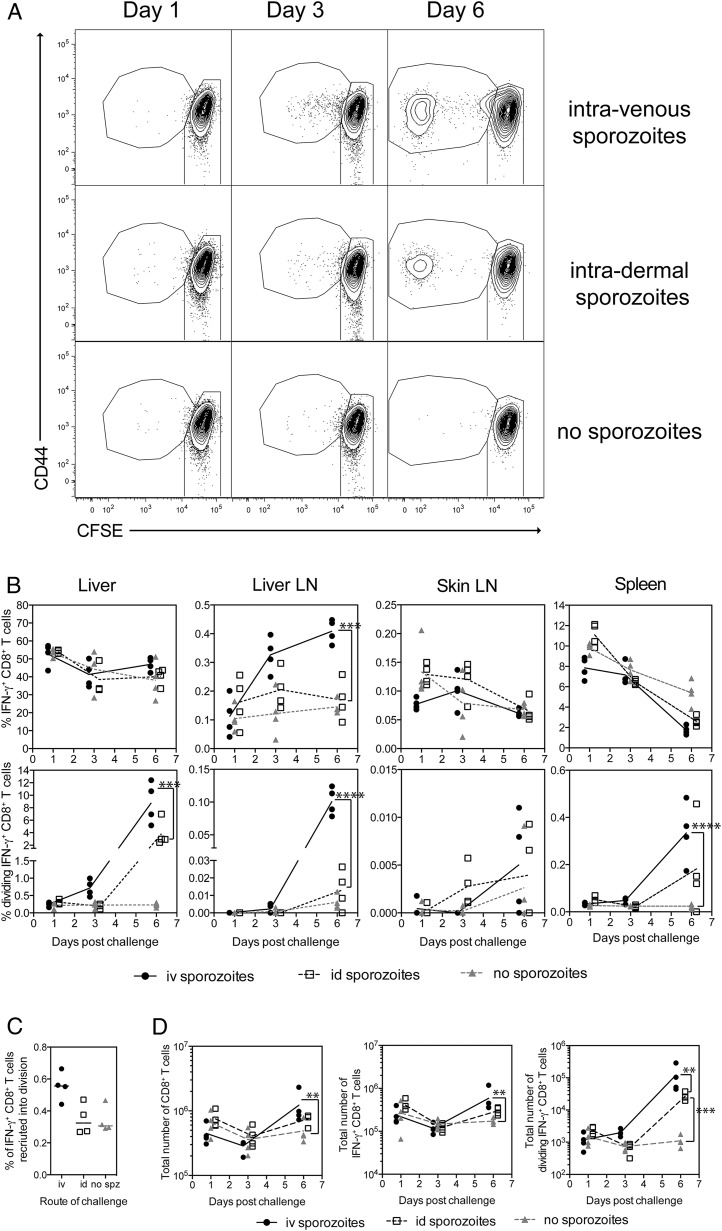
CD8^+^ T cells do not actively migrate into the liver during sporozoite infection. Pb9^+^CD8^+^ CFSE-labeled splenocytes (3 × 10^6^) from HAd5-MVA–vaccinated mice were transferred into BALB/c recipient mice 1 d prior to i.v. or i.d. challenge with 1000 *P. berghei* sporozoites. Livers, liver-draining lymph node (celiac and portal), skin-draining lymph nodes (auricular), and spleens were harvested at 1, 3, and 6 d for analysis of the Pb9 response by flow cytometry. Cells were surface stained with Live/Dead Aqua, CD4–eFluor 650, CD8-PerCP-Cy5.5, CD44–Alexa Fluor 700, CD62L-PE-Cy7, CD127-allophycocyanin-Cy7, and CCR7–bi av-qDot 565 and stained intracellularly with TNF-α–PE and IFN-γ–eFluor 450. (**A**) Representative dot plots show CD44 versus CFSE expression of all CD8^+^ T cells isolated from the liver after sporozoite challenge. Owing to the low frequency of dividing cells observed at day 1 and day 3, the Pb9, GFP, and media fcs file from each mouse on each day were pooled together (concatenated) for presentation. (**B**) Graphs represent the frequency of Pb9 cells as a percentage of total CD8^+^ T cells (top panel) or frequency of dividing Pb9 cells (bottom panel) as a percentage of total CD8^+^ T cells present in the liver, liver-draining lymph, skin-draining lymph node, and spleen over time. (**C**) The graph represents the percentage of donor Pb9-specific cells that were recruited into division. (**D**) Graphs represent the total number of CD8^+^ T cells (left), IFN-γ^+^ Pb9-specific CD8^+^ T cells (middle), or dividing IFN-γ^+^ Pb9-specific CD8^+^ T cells (right) in the liver over time. Individual animals are displayed as a single point, and the mean per group is represented by the line. Total cell numbers were log transformed for analysis. Each data set was analyzed with a two-way ANOVA and post hoc Bonferoni multiple comparison test to compare between groups at each time point. **p < 0.01, ***p < 0.001, ****p < 0.0001.

To determine whether the increase in IFN-γ^+^ and dividing CD8^+^ T cells was the result of active migration of CD8^+^ T cells into the liver in the presence of sporozoites (as opposed to fluctuations in cell numbers by performing liver preparations and peptide stimulations on different days), 3 × 10^6^ Pb9-specific splenocytes were transferred into naive mice prior to challenge via the i.v. or i.d. route of injection, while one group of mice remained unchallenged. On days 1, 3, and 6 postinfection, liver, liver-draining lymph nodes (celiac and portal), skin-draining lymph nodes (auricular), and spleens were harvested and the immune response was measured by ICS. Consistent with the previous experiment, dividing Pb9-specific cells were only observed in the liver of challenged mice on day 3 and day 6 after sporozoite challenge (Fig. 2A). No significant difference in the frequency of Pb9-specific cells in the liver was observed between all three groups after sporozoite administration (Fig. 2B), whereas a significant increase in dividing Pb9-specific cells was observed in the liver on day 6 in i.v. or i.d. challenged mice (Fig. 2C). A small but not statistically significant increase in the frequency of dividing Pb9^+^ T cells was observed at day 3 in i.v. challenged mice compared with i.d. or no sporozoite controls (Fig. 2C), suggesting again that fewer parasites reached the liver after i.d. versus i.v. administration. In the liver-draining lymph node, an increase in the frequency of Pb9-specific cells was observed after i.v. challenge at both day 3 and day 6 compared with unchallenged mice, with the largest increase observed in the frequency of dividing Pb9-specific cells at day 6 (Fig. 2C). Despite observing dividing cells in the liver at day 3, there was no difference between groups at day 3 in the liver-draining nodes or spleen, again suggesting that the liver is the main site of CD8^+^ T cell reactivation following i.v. sporozoite challenge. To our surprise, no change in either the frequency of Pb9-specific or dividing Pb9 cells was observed in the skin-draining lymph nodes (Fig. 2B, 2C); however, the frequency of Pb9 cells was extremely low. In two other experiments, dividing Pb9 specific cells were not observed in the blood at day 2 even when dividing cells were observed in the spleen the following day (Supplemental Fig. 3A), and only a very small frequency of dividing donor Pb9 cells was observed in the spleen at day 3 when a large proportion of dividing donor Pb9 cells was observed in the liver (Supplemental Fig. 3B). Although it has been reported that the spleen is the main site of naive CD8^+^ T cells activation after i.v. sporozoite administration (30, 45, 46), our data would suggest that the liver is the main site of effector CD8^+^ T cell reactivation.

In a separate experiment the phenotype of T cells was investigated in an attempt to find a predictive phenotype of dividing cells. On day 3, dividing Pb9^+^CD8^+^ T cells showed an increased CD69 and CXCR3 surface expression compared with the total population of Pb9^+^CD8^+^ T cells; they had a similar level of expression of CD44, CD62L, and CD103 (Supplemental Fig. 4). However, the pattern of surface marker expression would not suggest that dividing cells are a specialized population of cells, and it is more likely that division has resulted in a change in surface marker expression, as similar levels of CD69 and CXCR3 expression were observed on some undivided cells (Supplemental Fig. 4).

In summary, the data demonstrated that there was no active migration of large numbers of Ag-specific CD8^+^ T cells into the liver during the liver-stage malaria infection, the first 2 d after sporozoite challenge, when measuring responses at a total organ level. As dividing cells only appear after the parasites would have exited the liver, recruitment into division is not required for effector CD8^+^ T cells to mediate protection. However, recruitment into division does appear to be indicative of Ag presentation in the liver.

### CD8^+^ T cells require <24 h to mediate their protective effect

**FIGURE 3.**
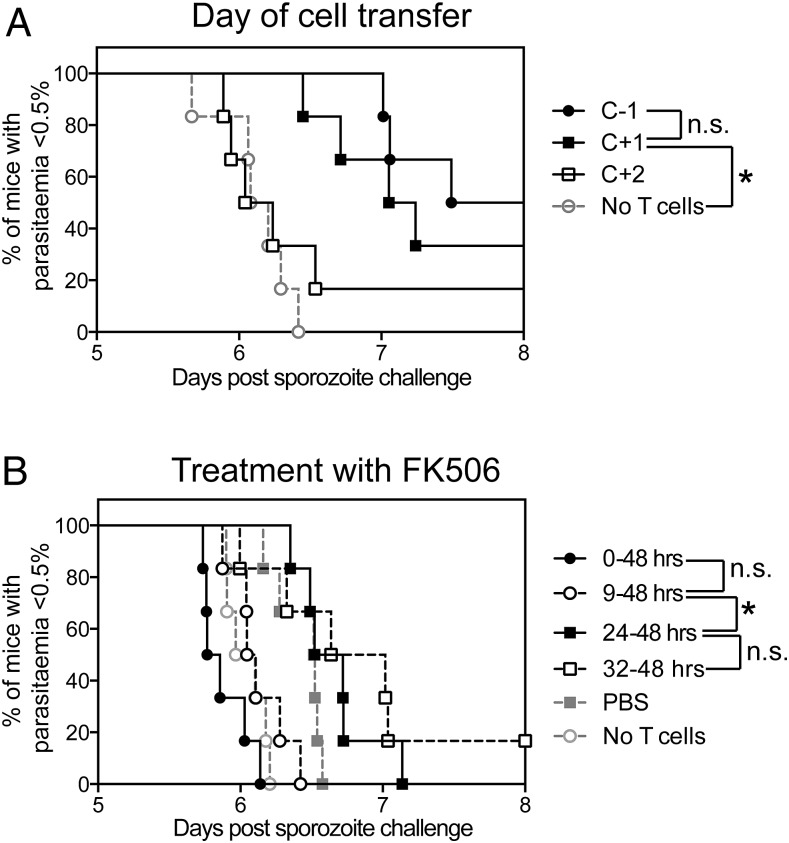
Pb9-specific cells only require 24 h to mediate a protective effect. (**A**) Pb9^+^ CFSE-labeled splenocytes (3 × 10^6^) from HAd5-MVA TIPeGFP–vaccinated mice were transferred into BALB/c recipient mice 1 d prior (C−1), 1 d after (C+1) or 2 d after (C+2) i.v. challenge with 1000 *P. berghei* sporozoites (*n* = 6 per group). Thin blood films were taken from day 5 onward to calculate the percentage of infected RBCs; the graph shows time to 0.5% parasitemia. Data are representative of two independent experiments. (**B**) Pb9^+^ CFSE-labeled splenocytes from HAd5-MVA TIPeGFP–vaccinated mice were transferred into BALB/c recipient mice 1 d prior to challenge. Mice were injected i.p. with 100 μl of PBS or 0.03 mg/ml FK-506 every morning and evening for the first 2 d postchallenge starting at the time of sporozoite injection (0–48 h) or from 9, 24, or 36 h postinjection (*n* = 6 per group). Survival curves were analyzed by log-rank (Mantel–Cox) test. An asterisk denotes a significant difference between groups after Bonferoni correction for the number of statistical tests performed on each data set. Data are representative of two independent experiments. *p < 0.05.

The previous experiments demonstrated that large numbers of Ag-specific CD8^+^ T cells were not actively recruited into the liver, and therefore we hypothesized that the need for a high number of circulating CD8^+^ T cells may be to ensure that a sufficient number of CD8^+^ T cells are present in the liver at the time of challenge to quickly identify and kill infected cells. To formally test this hypothesis, 3 × 10^6^ CFSE^+^Pb9^+^ T cells were transferred into recipient mice the day before challenge (as above) or 1 and 2 d after i.v. sporozoite challenge, and survival was measured as time to 0.5% parasitemia. A statistically significant delay in time to parasitemia was observed when Pb9 cells were transferred on the day before and the day after challenge (Fig. 3A) compared with no T cell controls. Although transferring cells the day before challenge appeared to improve survival, there was no statistical difference between groups, whereas transferring cells 2 d after challenge had no impact on protection compared with no T cell control mice.

As an alternative method to investigate the timing of CD8^+^-mediated protection, mice received 3 × 10^6^ Pb9-specific cells and i.v. sporozoites, and the immunosuppressive drug FK-506 (tacrolimus), which is reported to block MHC class I Ag presentation (35) but also has T cell immunosuppressive effects, was administered for varying lengths of time during the liver-stage infection period. FK-506 has a reported half-life of 12 h when administered via the i.v. route and therefore was administered in the morning and evening for the first 2 d after sporozoite administration. When FK-506 was administered at the same time as sporozoites, no delay in protection afforded by the transfer in Pb9-specific T cells was observed (Fig. 3B); if anything, mice succumbed to malaria faster than did naive control mice (but this was not statistically significant). By delaying treatment for half a day (9 h), a small nonsignificant delay in parasitemia was observed (Fig. 3B), but this was not different from naive control mice. A significant increase in survival was only observed when treatment was delayed by at least 24 h (Fig. 3B), with no significant improvement observed by delaying treatment for a further half a day. In summary, CD8^+^ T cells only require a short amount of time to mediate their effect, that is, <24 h. Taken together, the data would suggest that between 9 and 24 h is required for sporozoites to infect, Ag to be processed and presented, and CD8^+^ T cells to locate and kill infected hepatocytes.

### Reactivation of CD8^+^ T cells is dependent on sporozoite invasion of hepatocytes

**FIGURE 4.**
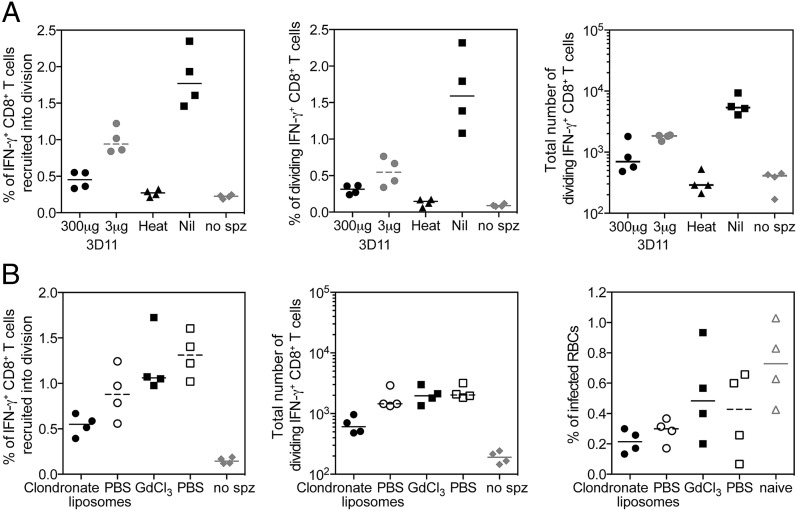
Division of CD8^+^ T cells is dependent on viable parasites invading hepatocytes. (**A**) Pb9^+^ CFSE-labeled splenocytes (3 × 10^6^) from HAd5-MVA TIPeGFP–vaccinated mice were transferred into BALB/c recipient mice 1 d prior to injection of 1000 sporozoites preincubated with 300 or 3 μg of 3D11 mAb, which had been heat inactivated at 56°C for 45 min, were untreated (Nil), or did not receive sporozoites. Livers were harvested 3 and 6 d later for analysis of Pb9-specific cells by flow cytometry. Pb9-stimulated cells were surface stained with Live/Dead Aqua, CD4-allophycocyanin–Alexa Fluor 780, CD8-PerCP-Cy5.5, CD44–Alexa Fluor 700, CXCR3-allophycocyanin, DX5-PE, and γδTCR-PerCP–eFluor 710 and stained intracellularly with IFN-γ–eFluor 450. Graphs represent the percentage of donor Pb9-specific cells that were recruited into division (left), the frequency of dividing Pb9 cells as a percentage of total CD8^+^ T cells (middle), and the total of Pb9-specific dividing CD8^+^ T cells (right) present in the liver on day 3. Data in each graph were analyzed with a Kruskal–Wallis and post hoc positive test to compare treatment groups; *p* < 0.05 was only observed between heat-inactivated versus Nil treatment or no sporozoites versus Nil treatment in each data set. Individual animals are displayed as single points, and the median per group is represented by the line (*n* = 4 per group). (**B**) Pb9^+^ CFSE-labeled splenocytes (3 × 10^6^) from HAd5-MVA TIPeGFP–vaccinated mice were transferred into recipient mice 1 d prior to injection of 1000 sporozoites. Recipient mice were injected i.v. with 200 μl of clodronate or PBS liposomes for 2 d prior to challenge (C−2 and C−1) or 100 μl of 2 mg/ml gadolinium chloride (GdCl_3_) or PBS the day before challenge (same time as cells). Individual animals are displayed as single points, and the median per group is represented by the line (*n* = 4 per group). Livers were harvested 3 and 6 d later for analysis of Pb9-specific cells by flow cytometry. Stimulated cells were surface stained with Live/Dead Aqua, CD4–eFluor 650, CD8-PerCP-Cy5.5, CD44–Alexa Fluor 700, CXCR3–allophycocyanin, and Vβ3-PE and stained intracellularly with IFN-γ–eFluor 450. Graphs represent the percentage of donor Pb9-specific cells that were recruited into division (left) and the total number of Pb9 specific dividing CD8^+^ T cells present in the liver on day 3 (middle) and parasitemia measured by thin blood film taken on day 6 (right). spz, sporozoite.

Sporozoites are known to infect Kupffer cells and traverse several hepatocytes (47–49) prior to asexual replication, in the process shedding surface proteins. Therefore, sporozoite Ags could be presented in the liver by a variety of cells, be they Kupffer cells that have phagocytized shed Ag or dead sporozoites or a hepatocyte that has been traversed or is the final infected cell. We wanted to determine whether Ag presentation in the liver and recruitment of Pb9-specific cells into division was impacted by the viability of the sporozoites and whether noninfectious sporozoites were an important source of Ag to assist in the reactivation of CD8^+^ T cells. Sporozoites were preincubated with the mAb 3D11 to reduce cell invasion or were heat killed by incubation at 56°C prior to injection into recipient mice. Reducing cell invasion by increasing the concentration of blocking 3D11 Ab led to a reduced number of donor Pb9-specific cells recruited into division and therefore reduced frequency and number of dividing cells detected in the liver of mice on day 3 and day 6 (Fig. 4A), although neither a dose of 300 or 3 μg was statistically significant from mice receiving untreated sporozoites. Injection of heat-killed sporozoites led to a significant decrease in the frequency of Pb9 cells recruited into division and the frequency of dividing Pb9 cells in the liver on both days 3 and 6 (Fig. 4A), which was not above the background level observed in unchallenged (no sporozoites) mice. Taken together, the data show that noninfectious sporozoites do not enhance or make a significant contribution to CD8^+^ T cell reactivation in the liver.

Kupffer cells are crucial to successful infection of mice with malaria (50) by creating a portal for sporozoites to invade hepatocytes. Given their close proximity to an infected cell, it is plausible that they may be involved in locally recruiting CD8^+^ T cells to the site of an infected hepatocyte; conversely, by presenting malaria Ags they could be misdirecting effector CD8^+^ T cells away from the infected cells. Therefore, we wanted to determine the contribution, be it positive or negative, of Ag presented by Kupffer cells to the reactivation and protection mediated by CD8^+^ T cells. Clodronate liposomes were administered for 2 d prior to sporozoite challenge to deplete Kupffer cells, or gadolinium chloride was administered the day before challenge to block phagocytosis. Reducing phagocytosis did not have a significant impact on Ag presentation, as a similar frequency of donor Pb9-specific cells were recruited into division in gadolinium chloride– and PBS-treated mice (Fig. 4B, left). Although administration of clodronate liposomes led to a reduced percentage of donor Pb9 cells recruited into division (Fig. 4B, left) and total number of dividing Pb9-specific cells detected in the liver on day 3 (Fig. 4B, middle), Pb9-mediated protection from malaria was not abolished in the absence of Kupffer cells, as a significantly lower level of parasitemia was detected in clodronate liposome–treated mice compared with naive control mice (Fig. 4B, right). Taken together, this would suggest that Kupffer cells are an important reservoir of parasite Ags but are not required for CD8^+^ T cells to mediate protection, because in their absence CD8^+^ T cells are still capable of locating and killing infected hepatocytes.

### Protection is dependent on a fine balance between the number of effector and target cells

**FIGURE 5.**
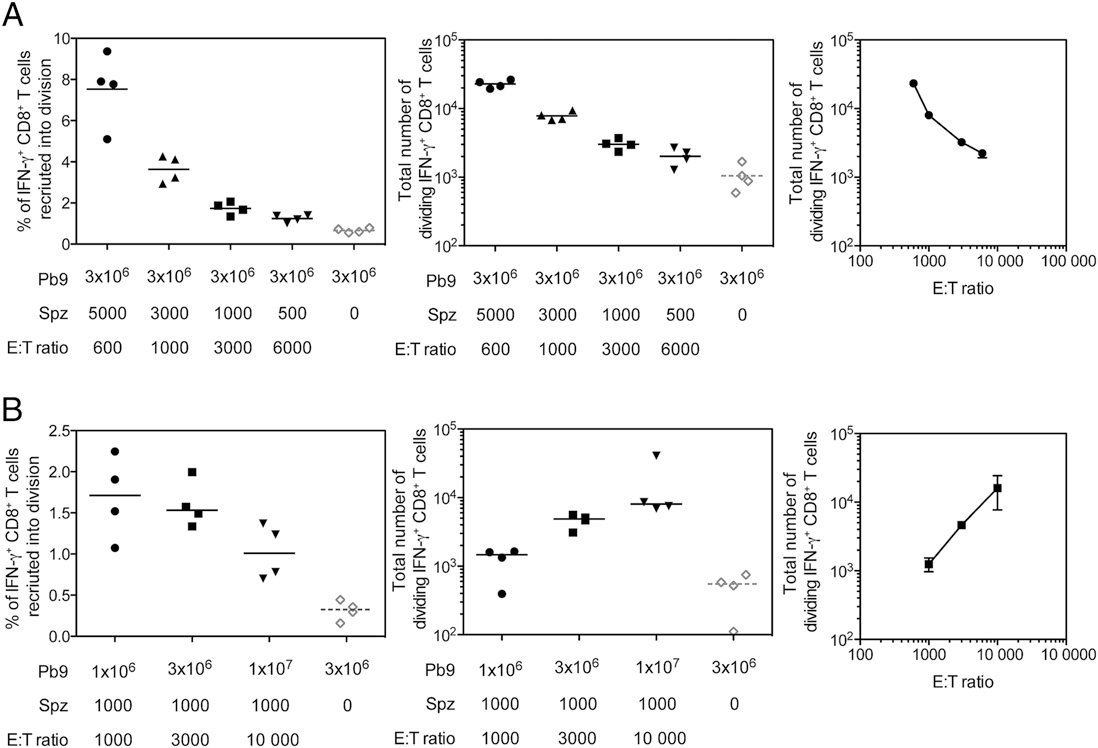
The affect of altering E:T ratios on dividing cell numbers. (**A**) CFSE-labeled splenocytes (3 × 10^6^ Pb9^+^) from HAd5-MVA TIPeGFP–vaccinated mice were transferred into BALB/c recipient mice 1 d prior to i.v. challenge with 5000, 3000, 1000, or 500 *P. berghei* sporozoites. Livers were harvested 3 d later for analysis by flow cytometry. Simulated cells were surface stained with Live/Dead Aqua, CD4–eFluor 650, CD8-PerCP-Cy5.5, CD44–Alexa Fluor 700, CXCR3-allophycocyanin, and Vβ3-PE and stained intracellularly with IFN-γ–eFluor 450. Graphs represent the percentage of Pb9-specific cells that were recruited into division (left), the total number of dividing Pb9-specific CD8^+^ T cells present in the liver on day 3 (middle), and the total number of dividing cells versus the E:T ratio (right). Each individual animal is displayed as a single point (*n* = 4 per group), and the mean per group is represented by the line; data are representative of two similar experiments. (**B**) Pb9^+^ CFSE-labeled splenocytes (1 × 10^6^, 3 × 10^6^, or 1 × 10^7^) from HAd5-MVA TIPeGFP–vaccinated mice were transferred into BALB/c recipient mice 1 d prior to i.v. challenge with 1000 *P. berghei* sporozoites. Livers were harvested 3 d later for analysis by flow cytometry. Stimulated cells were surface stained with Live/Dead Aqua, CD4–eFluor 650, CD8-PerCP-Cy5.5, CD44-CD62L-PE-Cy7, CD127-allophycocyanin-Cy7, and CXCR3-allophycocyanin and stained intracellularly with IFN-γ–e450. Graphs represent the percentage of donor Pb9-specific cells that were recruited into division (left), the total number of dividing Pb9 specific CD8^+^ T cells present in the liver on day 3 (middle), and the total number of dividing cells versus the E:T ratio (right). Individual animals are displayed as single points, and the median per group is represented by the line.

To further understand the relationship between circulating T cell numbers and Ag load on CD8^+^ T cell reactivation, we altered the E:T ratio by injecting different numbers of sporozoites or transferred Pb9 cells and measured the response in the liver 3 d postinfection. In the first experiment, the number of Pb9 cells remained constant (3 × 10^6^) when 5000 down to 500 sporozoites were injected, giving an E:T ratio range of 600–6000. As predicted, the number of donor Pb9 cells recruited into division decreased with the number of injected sporozoites (Fig. 5A, left). This resulted in a decreasing number of dividing Pb9 cells detected in the liver as the number of injected sporozoites decreased and the E:T ratio increased (Fig. 5A). In the second experiment, the number of injected sporozoites remained constant (1000 sporozoites) when 10^6^, 3 × 10^6^, or 10^7^ Pb9-specific cells were transferred, giving an E:T range of 1,000–10,000. No difference in the percentage of donor Pb9 cells recruited into division was observed when recipient mice received either 10^6^ or 3 × 10^6^ Pb9-specific cells. A small (albeit not significant) decrease in percentage of donor Pb9 cells recruited into division was seen when 10^7^ cells were transferred (Fig. 5B, left), but due to the higher number of cells injected, overall a higher number of dividing cells were present in the liver on day 3 (Fig. 5B, central). In contrast to the results observed in the first experiment, an increase in the number of dividing Pb9-specific cells was observed as the E:T ratio increased (Fig. 5B, right). If the number of dividing cells were simply a reflection of the number of infected cells (and thus Ag load), then we would predict that the number of dividing cells detected in the liver at day 6 would be constant regardless of the E:T ratio. The contrasting effect on dividing cell numbers by altering the E:T ratio in different ways, such that reducing the sporozoite numbers led to a decrease in dividing cells, but increasing transferred cells increased the number of dividing cells, would suggest that one of the primary factors influencing CD8^+^ T cell reactivation is the ability of cells to locate infected hepatocytes.

**FIGURE 6.**
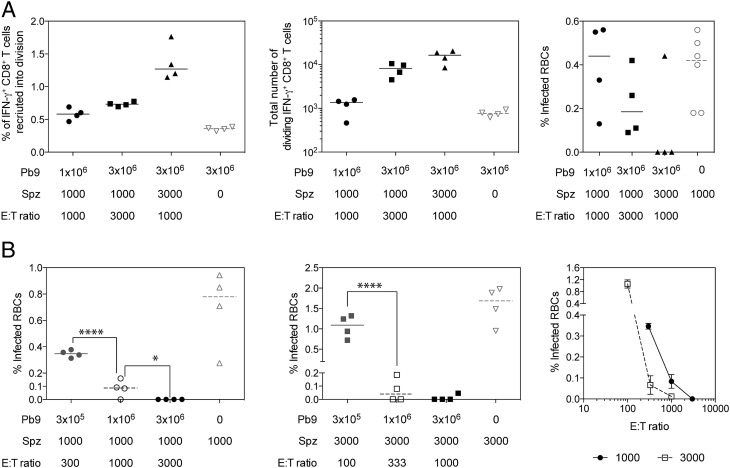
The effect of E:T ratio on sporozoite challenge outcome. (**A**) Pb9^+^ CFSE-labeled splenocytes (1 × 10^6^ or 3 × 10^6^) from HAd5-MVA TIPeGFP–vaccinated mice were transferred into BALB/c recipient mice 1 d prior to i.v. challenge with 1000 or 3000 *P. berghei* sporozoites. Livers were harvested 3 d later for analysis by flow cytometry or day six to measure parasitaemia. Stimulated cells were surface stained with Live/Dead Aqua, CD4–eFluor 650, CD8-PerCP-Cy5.5, CD44–Alexa Fluor 700, CD62L-PE-Cy7, CD127-allophycocyanin-Cy7, CXCR3-allophycocyanin, and CCR7–bi av-qDot565 and stained intracellularly with IFN-γ–eFluor 450 and TNF-α–PE. Each individual animal is displayed as single point (*n* = 4 per group), and the median per group is represented by the line. Graphs represent the percentage of donor Pb9-specific cells that were recruited into division (left), the total number of dividing Pb9 specific CD8^+^ T cells present in the liver on day 3 (middle), and parasitemia measured by thin blood film taken on day 6 (right). (**B**) Pb9^+^ CFSE-labeled splenocytes (3 × 10^6^, 1 × 10^6^, or 3 × 10^6^) from HAd5-MVA TIPeGFP–vaccinated mice were transferred into BALB/c recipient mice 1 d prior to i.v. challenge with 1000 or 3000 *P. berghei* sporozoites and mice were sacrificed at day 6 to measure Pb9-specific responses in the liver and parasitemia. Graphs represent the percentage of infected RBCs in mice challenged with 1000 (left) or 3000 (middle) sporozoites, or parasitemia related to the E:T ratio (right). Data in each graph was analyzed with a one-way ANOVA and post hoc positive test. *p < 0.05, ****p < 0.0001 between groups.

To further investigate the impact of the E:T ratio on protection, recipient mice received Pb9 T cells and sporozoites at an E:T ratio of 1000 or 3000, with the number of sporozoites and number of transferred Pb9 cells altered between groups. Consistent with previous experiments, increasing the number of transferred T cells or sporozoites injected increased the percentage of cells recruited into division (Fig. 6A, left) and total number of dividing cells (Fig. 6A, middle) detected in the liver on day 3. However, despite having the same E:T ratio, three of the four mice receiving 3 × 10^6^ Pb9 cells and 3000 sporozoites did not have any detectable parasites in the blood at day 6 compared with 10^6^ Pb9 cells and 1000 sporozoite recipient mice who were all infected on day 6. Although not statistically significant, the percentage of infected RBCs on day 6 decreased as the number of transferred Pb9 cells and sporozoite increased (Fig. 6A, right), even when the E:T ratio decreased as more sporozoites were injected.

To further explore the relationship between the E:T ratio and protection, BALB/c mice received 3 × 10^5^, 1 × 10^6^, or 3 × 10^6^ Pb9^+^ splenocytes prior to challenge with either 1000 or 3000 *P. berghei* sporozoites. Consistent with the previous experiment and published data, transferring an increasing number of Pb9 cells resulted in lower levels of parasitemia at day 6 (Fig. 6B), and this was true when mice were challenged with 1000 or 3000 sporozoites. In mice that received 3 × 10^5^ Pb9 cells, those challenged with 3000 sporozoites had significantly higher levels of parasitemia when compared with mice challenged with only 1000 sporozoites (Fig. 6B). In contrast, when higher numbers of Pb9 cells were transferred, mice challenged with 3000 sporozoites had equivalent levels of parasitemia compared with mice receiving the same number of cells, but challenged with 1000 sporozoites (Fig. 6B). Once again, despite maintaining the same (1000) or a similar (300 compared with 333) E:T ratio, mice challenged with 3000 sporozoites had lower levels of parasitemia at day 6 (Fig. 6B, right). The data demonstrate that although the E:T ratio is important, it cannot be used as a predictor for protection, as when there were higher numbers of effector cells and parasites, more CD8^+^ T cells were able to locate and kill the infected sporozoites in the 2 d that parasites were present in the liver. Taken together, the data demonstrate the fine balance of CD8^+^ T cell–mediated protection from liver-stage malaria, where protection is dependent on having enough CD8^+^ T cells circulating through the liver and enough hepatocytes infected to reactivate CD8^+^ T cells.

## Discussion

Although CD8^+^ T cells have been shown to play a critical role in protection from liver-stage malaria, very little is known about the kinetics of CD8^+^ T cell reactivation to sporozoite challenge and its impact on protection. By transferring Ag-specific effector CD8^+^ T cells from a vaccination regimen capable of inducing CD8^+^-mediated protection (51), we developed an adoptive transfer system to identify and understand the critical elements involved in CD8^+^ T cell–mediated protection from liver-stage malaria.

Although intravital imaging studies have demonstrated CD8^+^ T cell clusters around infected hepatocytes (52, 53), to our surprise, no active migration of large numbers of effector CD8^+^ T cells was observed at a macroscopic whole-liver level (Figs. 1, 2), as fluctuations in CD8^+^ T cell numbers were not Ag specific (Fig. 1) or due to sporozoite challenge (Fig. 2). Therefore, detailed intravital microscopy studies will be necessary to identify the mechanism by which CD8^+^ T cells kill infected hepatocytes. By transferring CFSE-labeled cells, we were able to show that only 1% of the donor Pb9-specific CD8^+^ T cells in the liver recognized Ag and were subsequently recruited into cell division (Figs. 2, 4–6). In contrast, very few (if any) dividing cells were detected in the liver-draining lymph nodes or spleens (Figs. 1, 2, Supplemental Fig. 3), suggesting that the liver is the main site of effector T cell reactivation following i.v. sporozoites. These data are in contrast to activation of primary CD8^+^ T cells in response to i.v. irradiated sporozoites where priming of naive CD8^+^ T cells occurs primarily in the spleen or draining lymph nodes (29, 30, 46). Given that activation of naive CD8^+^ T cells in the liver induces an abortive or tolerized immune response (54, 55), it is not surprising that the main site of activation of naive versus effector CD8^+^ cells is different for sporozoite Ags. The sudden appearance of divided Pb9-specific cells observed in this study is consistent with reports of CD8^+^ memory T cells mediating effector functions quickly, but being slow to start dividing, with dividing cells rapidly progressing through multiple rounds of cell division once division is initiated (56). As dividing cells were only detected after parasites would have exited the liver, the data clearly demonstrate that recruitment into division is not required for CD8^+^ T cells to mediate protection, but division is merely in response to recognition of Ag.

Despite the low frequency of responding cells, we have been able to demonstrate that recruitment into division can be used as a surrogate marker of Ag presentation in the liver. Injection of higher numbers of sporozoites led to an increase in the percentage of cells recruited into division (Fig. 5) in a dose-dependent manner. Reduced Ag presentation was observed when sporozoites were injected i.d. compared with i.v. injection (Fig. 2, Supplemental Fig. 4), consistent with previous reports showing reduced parasite levels in the liver after i.d. injection of sporozoites (57). The data also demonstrated that although Kupffer cells are an important source of sporozoite Ags, they are not critical for Ag presentation in the context of protection because depletion of Kupffer cells reduced recruitment into division but it did not impact CD8^+^ T cell–mediated protection (Fig. 4). These data are supported by previous evidence from bone marrow chimera mice in which CD8^+^ T cell–mediated protection was lost when hepatocytes did not express the correct MHC to be able to present sporozoite Ag to effector cells (25). Of most interest, the data demonstrated that the level of Ag presentation in the liver is dependent on the dose of infectious parasites. Significantly reduced recruitment into division in the liver of mice was observed when sporozoites were heat killed prior to injection, consistent with a previous report (58). Additionally, reducing sporozoite entry into hepatocytes by the addition of blocking mAb substantially reduced the number of dividing cells present in the liver (Fig. 4), consistent with reports of reduced Ag presentation in skin-draining lymph nodes after i.d. administration of sporozoites and blocking mAbs (29). The inability on noninfectious sporozoites to induce sufficient CD8^+^ T cells responses may also explain why such high doses of cryopreserved irradiated sporozoites are required to achieve protection in humans (59) compared with those administered via live mosquito bite (60). Importantly, it has only been through direct venous inoculation of irradiated sporozoites that 100% efficacy has been observed, with higher CD8^+^ T cell responses detected in protected versus infected volunteers (61). Collectively, the results suggest that vaccines aimed at inducing sporozoite blocking Abs could have a negative effect on boosting liver-stage CD8^+^ T cells upon malaria exposure, as there will be a reduced level of Ag presentation in the liver.

This finding has potential relevance for the development of combination vaccine approaches for malaria. Current models of the interaction of anti-sporozoite and anti–liver-stage vaccines (62) assume independent effects of these components and conclude that substantial additive or superadditive efficacy may be observed. This prediction is currently being assessed in phase IIa trials (T. Rampling, K.J. Ewer, G. Bowyer, N.J. Edwards, D. Wright, S. Sridhar, R. Payne, J. Powlson, C. Bliss, N. Venkatraman, I.D. Poulton, E. de Barra, H. de Graaf, A. Grobellar, H. Davies, R. Roberts, K. Ivinson, R. Weltzin, B.-Y. Rajkumar, U. Wille-Reece, C. Lee, C. Ockenhouse, R.E. Sinden, S. Gerry, A. M. Lawrie, J. Vekemans, D. Morelle, M. Lievens, R.W. Ballou, D. Lewis, G.S. Cooke, S.N. Faust, S. Gilbert, and A.V.S. Hill, manuscript in preparation). However, if one consequence of reducing sporozoite numbers is that T cells are less well activated in the liver, additive effects might be lower than predicted. Another implication of our findings is that if chronic exposure to repeated sporozoite infections leads to persistence of Ag in the liver in hyper-endemic regions, then reactivation of specific T cells may be facilitated. Interestingly, the liver-stage ChAd63-MVA ME-TRAP vaccine candidate that showed just >20% sterile efficacy in immunologically naive U.K. subjects (23) showed a higher efficacy of 67% against natural infection in semi-immune Kenyan adults (63). Several factors could contribute to this difference, including a greater level of persistent Ag in the liver in semi-immune individuals.

Although the duration of the liver-stage infection in mice is short (7), we show in this study that when CD8^+^ T cells identify infected hepatocytes, they require <24 h to mediate their effector function (Fig. 3). Transferring CD8^+^ T cells into infected mice 24 h after sporozoite injection could still reduce the time to 0.5% parasitemia (Fig. 3), consistent with a previous in vivo study (52) and with our in vitro killing assay where a reduction in the number of sporozoite-infected hepatocytes can be observed within 24 h after the addition of CD8^+^ T cells (64). When administration of FK-506 was used to block Ag presentation and T cell function, reduced time to parasitemia was observed when the drug was administered 24 h but not 9 h into the infection. As optimal Ag presentation by hepatocytes is observed in vitro after 8 h of sporozoite infection (65), we hypothesize that the time limit we observed in vivo is a combination of the time required for processing and presentation of sporozoite Ags and CD8^+^ T cells to mediate their effect.

In this study, by titrating the number of sporozoite and effector CD8^+^ T cells we discovered a threshold of protection dependent on both having enough infected hepatocytes and enough effector CD8^+^ T cells circulating through the liver. In contrast to lymph nodes that have a relatively simple draining pattern to ensure T cells can quickly scan APCs, livers are large multilobular organs with extensive circulatory systems and thus effector T cells may require several passes through the liver to locate the infected cells. Therefore, it is not surprising that it is easier to stimulate a recall CD8^+^ T cell response when there is a higher number of infected hepatocytes. Accordingly, unlike a simple in vitro assay (or T cell activation in a lymph node), in vivo protection cannot easily be predicted by the E:T ratio. In all experiments, only a small percentage of donor Pb9-specific CD8^+^ T cells recognized peptide–MHC on the infected hepatocytes and were subsequently recruited into cell division, typically 1% of donor Pb9-specific cells (Figs. 1C, 5, 6). To achieve protection in mice, >10^4^ reactivated CD8^+^ T cells were required (Figs. 4B, 6A), which equates to ∼10^6^ Ag-specific CD8^+^ T cells that are required in the liver during a liver-stage infection to ensure protection, consistent with previous reports (53). Because the liver stage of malaria infection in humans is longer, providing CD8^+^ T cells with more time to locate infected hepatocytes, we would predict that the threshold of protection, in terms of number of CD8^+^ T cells required, will be lower in humans, as suggested by recent clinical data (23).

In the last decade, vaccination with a heterologous combination of viral vectors ChAd63 and MVA expressing a single *P. falciparum* Ag TRAP has achieved high numbers of circulating CD8^+^ T cells in mice, primates, and humans (51, 66, 67). With an increase in the number of TRAP-specific cells induced, partial efficacy in malaria naive and exposed individuals can now be achieved (23, 63). One strategy to increase the number of Ag-specific CD8^+^ T cells has been the inclusion of molecular adjuvants that have shown some success in mice and primates (51). Given the small number of infected hepatocytes, a more successful approach is likely to be inclusion of several newly identified liver-stage Ags (68) together in one vaccine, or novel vaccination strategies to target T cells to the liver (A. Gola, A.A. Walters, A.M. Salman, B.R. Halbroth, S.M. Khan, C.J. Janse, R.N. Germain, A.J. Spencer, and A.V.S. Hill, submitted for publication). Given the fine balance in T cell–mediated protection observed in this system, multiple approaches will need to be investigated to ensure that next-generation vaccines induce CD8^+^ T cell responses above the threshold required for protection against liver-stage malaria.

## Supplementary Material

Data Supplement
