# Supplementary material for: The Threshold of Protection from Liver-Stage Malaria Relies on a Fine Balance between the Number of Infected Hepatocytes and Effector CD8+ T Cells Present in the Liver
Source: J Immunol. 2017 Jan 13;198(5):2006–16. doi: 10.4049/jimmunol.1601209 (PMC5318841; doi:10.4049/jimmunol.1601209)
Supplement: Data Supplement [file JI_1601209.zip › JI_1601209_Supplemental_Figures_1.pdf]

Spencer et al Supplementary Data

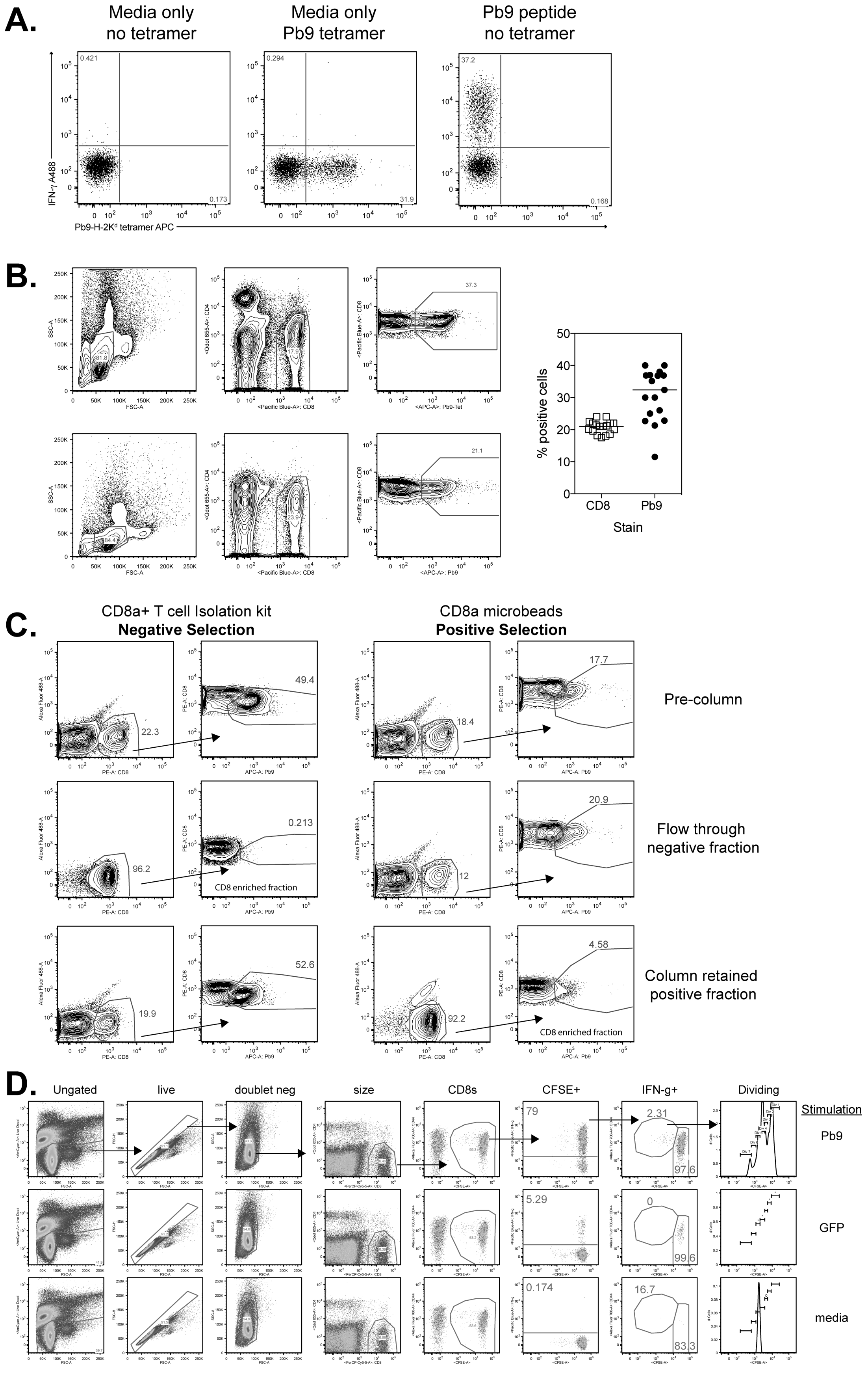

Figure S1: Adoptive transfer of Pb9 specific splenocytes and flow cytometry gating strategy

A. Blood samples from HAd5.TIPeGFP vaccinated mice were taken 14 days post vaccination and stimulated overnight with Pb9 peptide (or media) followed by Pb9 tetramer surface and intracellular IFN-γ staining. Dot plots show Pb9 tetramer staining vs intracellular IFN-γ on $CD8^+CD4^-$ lymphocytes.

B. Prior to transfer into recipient mice, splenocytes were stained with CD4, CD8 and Pb9 tetramer to calculate the number of total splenocytes to inject to achieve transfer of $3x10^6$ Pb9 specific $CD8^+$ T cells. Dot plots show the staining from 2 separate experiments, while graphs demonstrate the frequency of $CD8^+$ and $Pb9^+$ (as percentage of $CD8^+$ cells) from 17 different adoptive cell transfers.

C. On two separate occasions, splenocytes were enriched for $CD8^+$ T cells by either negative (Miltenyi Biotec $CD8a^+$ T Cell Isolation Kit over a LD column) or positive (CD8a (Ly-2) microbeads and LS column) selection prior to sorting for Pb9 tetramer positive cells on a MoFlow Cell Sorter. In both instances the CD8 enrichment was successful, the majority of $Pb9^+$ tetramer positive cells were observed within the CD8 negative fractions.

D. *Ex vivo* liver, lymph node and spleen cells were stimulated with the Pb9 or EGFP peptides for 6 hours and rested in the fridge overnight prior to surface and intracellular staining and data acquisition on a LSR II. Pb9 and GFP specific cells were identified by excluding dead cells with a FSC-Live-Dead aqua gate, removing doublets with a FSC-Area vs FSC-Height gate and gating on lymphocytes based on size with a FSC-Area vs SSC-Area gate. This was followed with gating for $CD8^+CD4^-$ cells and identifying donor cells based on CFSE positivity vs a CD44 gate.

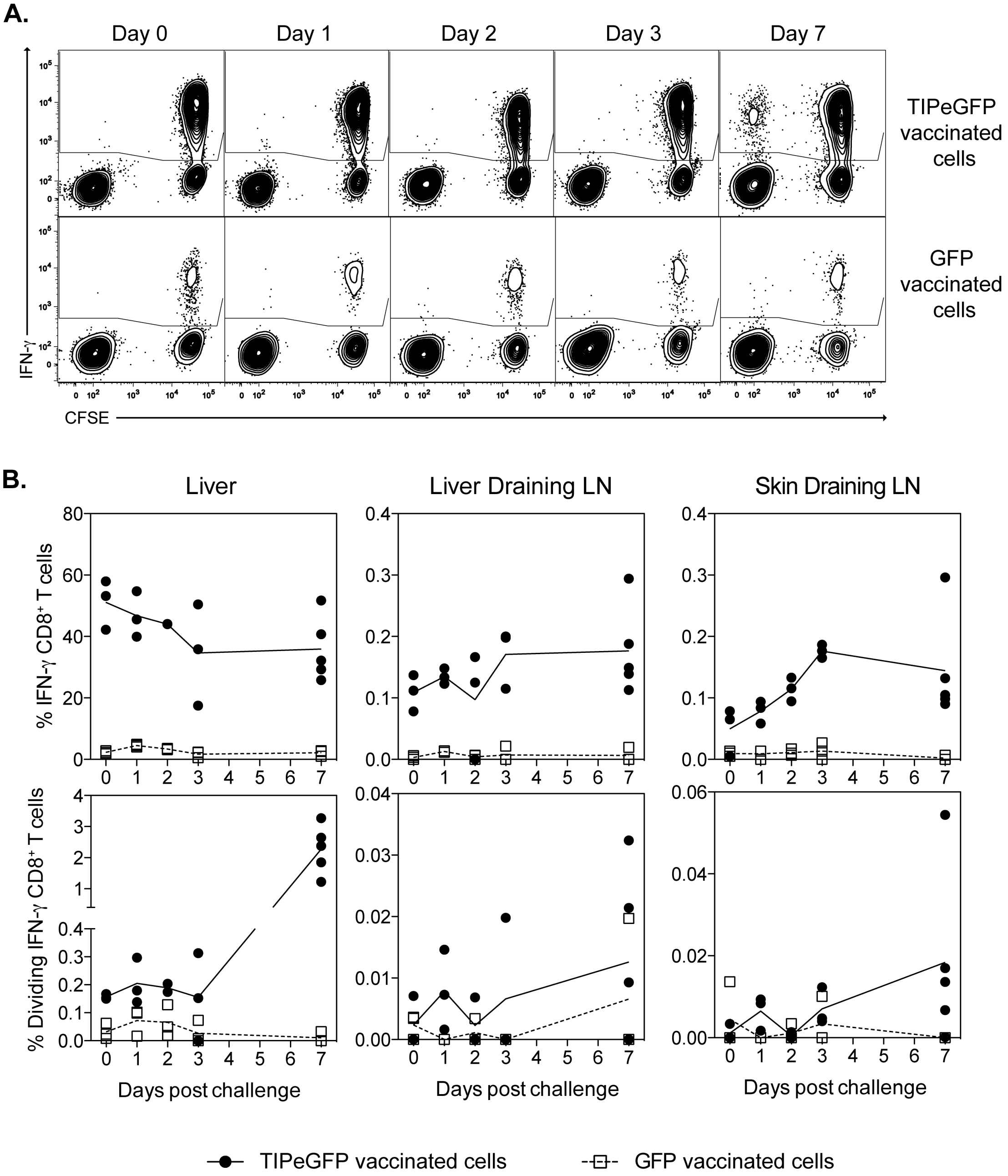

Figure S2: Kinetics of the CD8$^+$ T cell response to intradermal sporozoites

1x10^7 CFSE labeled splenocytes from HAd5-MVA TIPeGFP or GFP vaccinated mice were transferred into BALB/c recipient mice 1 day prior to intradermal challenge with 1000 *P.berghei* sporozoites. Mice were sacrificed on the day of challenge (Day 0) and 1, 2, 3 and 7 days post challenge to analyse the response in the livers, liver draining lymph nodes (celiac), skin draining lymph node (auricular) and spleen by flow cytometry. Stimulated cells were surface stained with LIVE/DEAD Aqua, CD8-Pacific Blue, CD62L-

PerCPCy5.5, CD127-bi av-qDot 565, CD4-e650, CD25-Alexa700, CD44-APCCy7 and stained intracellularly for IL-2-PE, TNF-α-APC and IFN-γ-e450.

Representative dot plots (**A**) show IFN-γ vs CFSE expression of all $CD8^+$ T cells isolated from the liver after sporozoites challenge. Due to the low frequency of dividing cells observed, the Pb9, GFP and media fcs file from each mouse were pooled together (concatenated) for presentation.

Graphs represent the frequency of IFN-γ^+ $CD8^+$ T cells (top graphs) or dividing IFN-γ^+ $CD8^+$ T cells from Pb9 (black filled circle) or GFP (open squares) vaccinated donor mice present in the liver, liver-draining lymph nodes or skin draining lymph nodes over time. Values are after background subtraction of the media stimulated wells, with each animal displayed as a single point and the median per group represented by the line.

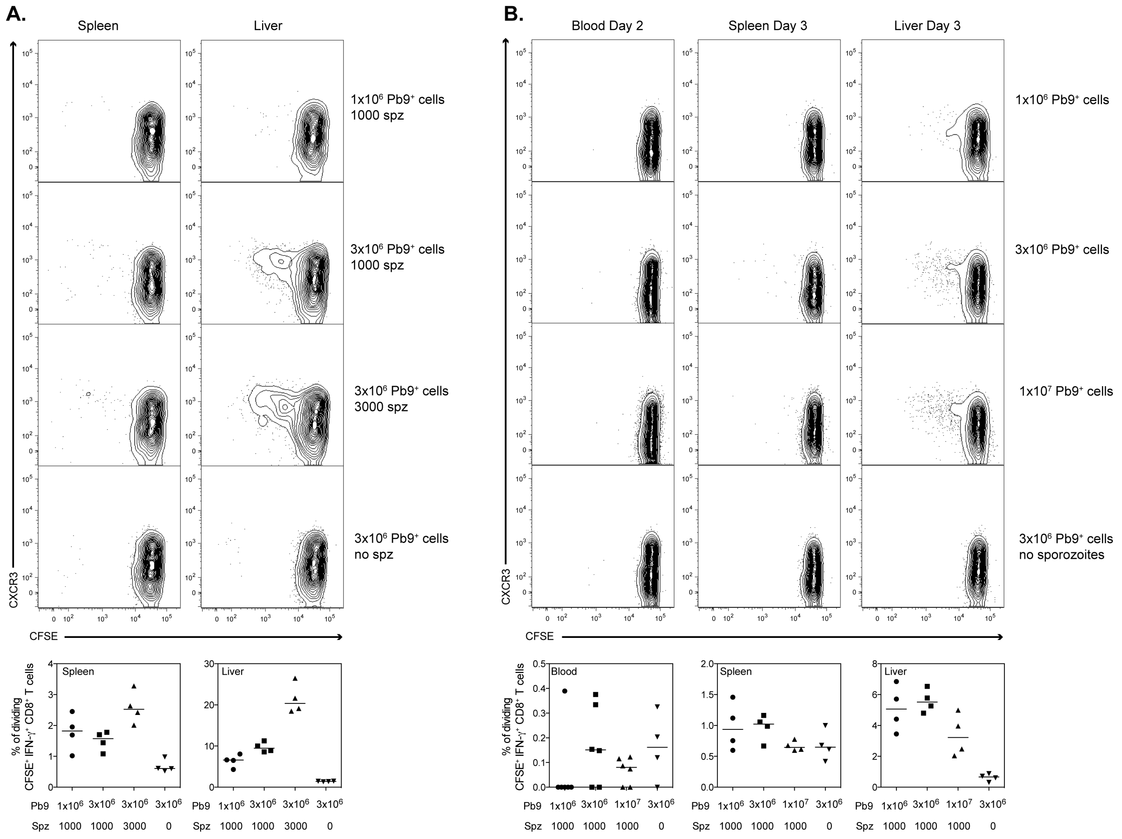

Figure S3: The highest frequency of dividing Pb9 specific cells are found in the liver and not blood, draining lymph nodes or spleens

A. In the same experiment as described in Figure 6A, representative dot plots show CFSE (x-axis) vs CXCR3 expression (y-axis) on all IFN-γ$^+$ CD8$^+$ T cells in Pb9 stimulated spleen and liver samples. Graphs represent the percentage of dividing cells as a frequency of donor Pb9 cells, lines represent the median per group with each individual mouse displayed as a single point.

B. CFSE labeled splenocytes containing 3x10^6 Pb9 specific cells from HAd5-MVA vaccinated mice were transferred into BALB/c recipient mice 1 day prior to intravenous challenge with 1000 *P.berghei* sporozoites. 2 days after challenge mice were bled and cells stimulated for ICS analysis. On day 3 a separate group of mice were sacrificed with spleens and livers harvested and cells stimulated for ICS analysis. Representative dot plots show CFSE (x-axis) versus CXCR3 expression (y-axis) on IFN-γ$^+$ CD8$^+$ T cells in stimulated blood, spleen and liver samples. Due to the low frequency of cells in blood samples all

the samples in a group were concatenated for display, while plots of the spleen and liver is a representative mouse per group. Graphs represent the percentage of dividing cells as a frequency of donor Pb9 cells, lines represent the median per group with each individual mouse displayed as a single point.

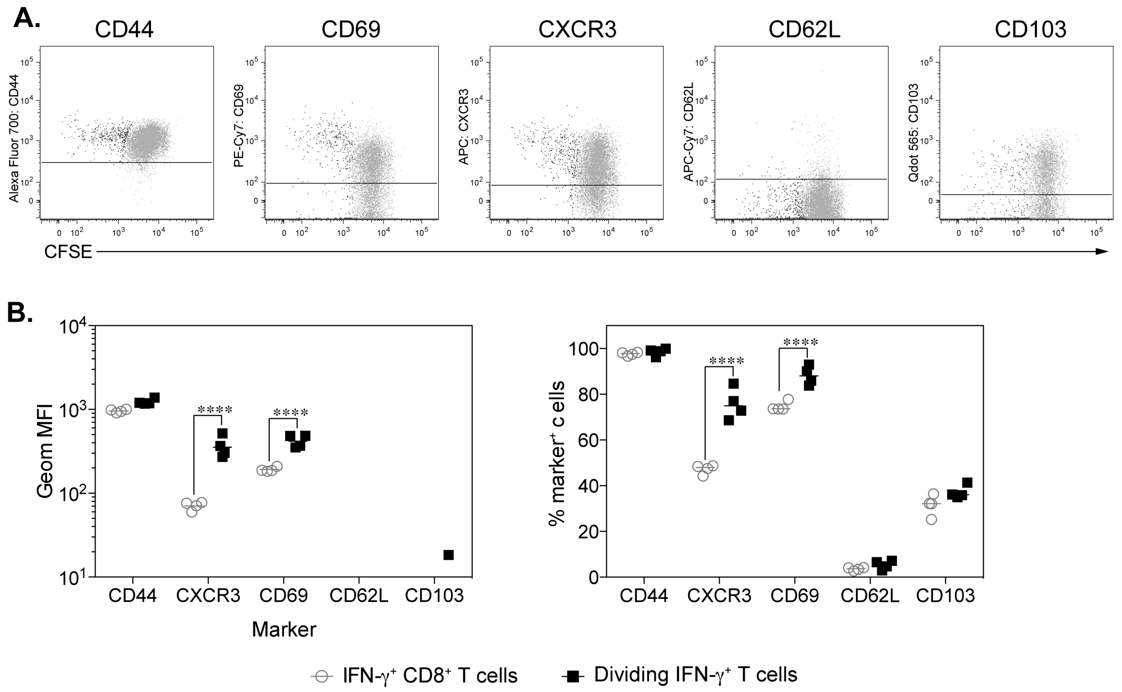

Figure S4: Phenotype of dividing cells

3x10[6] Pb9+ CD8+ CFSE labeled splenocytes from HAd5-MVA vaccinated mice were transferred into BALB/c recipient mice 1 day prior to intradermal challenge with 1000 *P.berghei* sporozoites. Livers were harvested 3 days later for analysis by flow cytometry. Stimulated cells were surface stained with Live-Dead Aqua, CD4-e650, CD8-PerCPCy5.5, CD44-Alexa700, CD62L-PECy7, CD127-APCCy7 and CCR7-bi av-qDot 565 and stained intracellularly with TNF-α-PE and IFN-γ-PB. Pb9 specific IFN-γ producing $CD8^+$ T cells were identified by gating on LIVE/DEAD negative, size (FSC-H vs SSC), doublet negative (FSC-H vs FSC-A), $CD8^+CD4^-$ cells and IFN-γ^+.

A. Dot plots show marker expression vs CFSE expression of all IFN-γ^+ $CD8^+$ T cells (grey) and dividing IFN-γ^+ $CD8^+$ T cells isolated from the liver after sporozoites challenge. Due to the low frequency of dividing cells observed for each animal, fcs files from samples stimulated with Pb9 were pooled together (concatenated) for presentation.

B. The graph represents the Geometric Mean Florescence Intensity (MFI) of each surface marker, individual animals are displayed as single points and the line represents the median per group. Negative

values for CD62L and CD103 are not shown. Data was log transformed and analysed with a 2-way repeated measure anova and post-hoc sidaks multiple comparison test to compare changes in marker expression between IFN-γ^+ and dividing IFN-γ^+ cells, **** denotes $p<0.0001$.

C. The graph represents the percentage of positive cells for each surface marker, individual animals are displayed as single points and the line represents the median per group. Data was analysed with a 2-way repeated measure anova and post-hoc sidaks multiple comparison test to compare changes in marker expression between IFN-γ^+ and dividing IFN-γ^+ cells, **** denotes $p<0.0001$.
